# Over-expression, Rapid Preparation and Some Properties of C-terminal BARc Region in PICK1

**DOI:** 10.3390/ijms10010028

**Published:** 2008-12-27

**Authors:** Hong Xiao, Yawei Shi, Jingming Yuan, Yuming Huang, Junhua Wang

**Affiliations:** 1 Department of Pathology, The First Affiliated Hospital, Shanxi Medical University, Taiyuan 030001, P.R. China; 2 Key Laboratory of Chemical Biology and Molecular Engineering of Education Ministry, Institute of Biotechnology, Shanxi University, Taiyuan 030006, P.R. China; 3 Jiangsu Kingsley Group Co., Nanjing 210000, P.R. China

**Keywords:** PICK1, BARc, human rhinovirus 3C protease cleavage, ammonium sulfate precipitation, inter-molecular interaction

## Abstract

A DNA fragment encoding C-terminal BARc region (amino acids 128–416) of rat PICK1 (NP_445912 ) was inserted into a modified vector pMAL-s involving human rhinovirus 3C protease cleavage site to produce a recombinant plasmid, pMAL-s-*barc*. The construct can express the fusion protein, MBP-BARc in the soluble form in *E.coli*. To remove the MBP tag, MBP-BARc purified from amylose beads was digested with human rhinovirus 3C protease and the cleavage efficiency is about 95% when the ratio of protein / enzyme (w/w) reaches 50:1, as analyzed on SDS-PAGE. The enzymatic reaction mixture was rapidly separated into two parts, MBP in the supernatant and BARc in the precipitate at the concentration of 1 M ammonium sulfate. In such case, the target protein BARc could be economically produced in a soluble state to be as the sample for measuring its biochemical function, for example, protein-protein interaction and protein-lipid combination.

## 1. Introduction

PICK1 (protein interacting with C kinase 1) is a protein kinase C (PKC) binding protein, initially identified by a yeast two-hybrid system [1]. It is a cytosolic protein containing a PDZ [postsynaptic density-95 (PSD-95)/Discs large/zona occludens-1] domain at N-terminus as well as a BAR (Bin/amphysin/Rvs) domain and an acidic amino acid region close to the C-terminus [2]. The PICK1 PDZ domain (amino acids 20-110) was shown to mediate the interaction with a broad range of proteins including receptor tyrosine kinases, ionotropic glutamate receptors of the l-α-amino-3-hydroxy-5- methyl-4-isoxazole propionic acid (AMPA) and kainate subtypes, metabotropic glutamate receptors, ion channels, G protein-coupled receptors, transmembrane transporters and ADP-ribosylation factors [2–5]. PICK1 BAR domain (amino acids 152–362) has an action of protein trafficking, especially endocytosis. The BAR domain of amphiphysin can directly bind and tubulate to liposomes [6]. It was reported that the amino acid sequence conservation among BAR domains from Endophilin, Amphiphysin and Arfaptin was quite low [6–8]. When the BAR domain is expressed in *E. coli*, the recombinant product frequently forms an aggregate. Therefore, the biochemical function of BAR domain has been rarely reported. In order to explore the biological function of BAR domain in rat PICK1 (NP_445912), we prepared a soluble fusion protein MBP-BARc and then, obtained the target protein BARc (amino acids 128–416). In this report, we constructed a new vector pMAL-s, which is modified from pMAL- p2X by substituting Factor Xa cleavage site with human rhinovirus 3C protease cleavage site. In such case, the expression product, MBP-BARc could be cleaved into MBP and BARc by the protease. However, it is still hard to separate the target product BARc from the enzymatic mixture by either ion-exchange chromatography or gel filtration due to its aggregate. After exploring some methods, an unexpected finding was that the BARc in the enzyme reaction mixture could be easily separated by ammonium sulfate precipitation. The finding provides a convenience for further studying the biological function of the BARc [9].

## 2. Materials and methods

### 2.1. Bacterial strains, plasmid and reagents

*E.coli* DH5α, *E.coli* JM109 and *E.coli* BL21 strains stored in this laboratory were used as host cells for cloning and expression. In addition, a recombinant strain was used to produce human rhinovirus 3C protease. Vector pMAL-p2X, amylose beads and restriction enzymes were all from New England Biolabs (Beverly, MA, USA). Bovine brain lipid extracts (Type I, Folch Fraction I,) was from Sigma-Aldrich Co. (St. Louis, MO, USA). The recombinant plasmid pRK5-*pick1* was a gift from Dr. MJ. Zhang (Department of Biochemistry, HKUST). An anti-BARc polyclonal antibody used was prepared by this research group. The human rhinovirus 3C protease was expressed and purified as described in reference [10]. The secondary antibody, rabbit anti-guinea pig immunoglobulin G conjugated horseradish peroxidase (HRP) was purchased from Boda Biotech. Co. (Beijing, P.R. China). All other chemicals used were commercially available.

### 2.2. Construction of recombinant plasmid pMAL-s-barc

**Figure 1.**
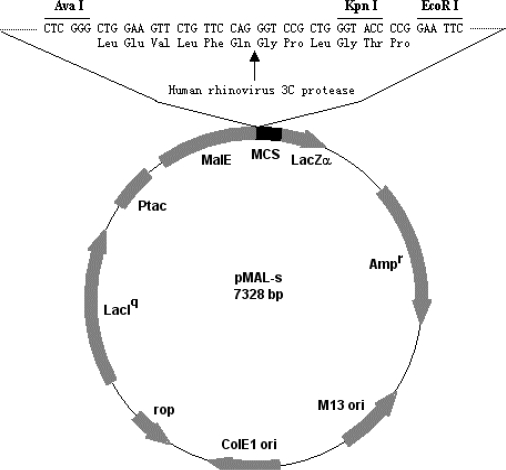
A diagrammatic map of pMAL-s. The Factor Xa cleavage site in pMAL-p2X is replaced with human rhinovirus 3C protease cleavage site.

A pair of chemically synthesized oligo-nucleotides containing human rhinovirus 3C protease cleavage site (5′-**CTCGGG**CTGGAAGTTCTGTTCCAGGGTCCGCTGGGTACCCCGG-3′, and 5′-**GAATTC**CGGGGTACCCAGCGGACCCTGGAACAGAACTTCCAGC-3′) was annealed. Then, the dsDNA oligo-nucleotide was inserted into pMAL-p2X vector where the Factor Xa cleavage site was already deleted via *EcoR*I and *Sal*I restriction sites to produce a modified vector pMAL-s. Therefore, the vector contains human rhinovirus 3C protease cleavage site (Figure 1). The gene sequence of the BARc was amplified by PCR with pRK5-*pick1* as the template in the presence of the forward primer 5′-CG**GAATTC**ATGAGTTCAGGCACAG-3′and reverse primer 5′-GGGG**GTCGAC**TCA- GGAGTCACACCAGC-3′ (*Eco*R I and *Sal* I sites are black). Finally, the PCR product was cloned into pMAL-s vector via *Eco*R I and *Sal* I restriction sites to generate the recombinant plasmid pMAL-s-*barc*. Both pMAL-s and pMAL-s-*barc* plasmids were verified by DNA sequencing.

### 2.3. Expression and purification of fusion protein, MBP-BARc

*Escherichia coli* strain JM109 was transformed with pMAL-s-*barc* and grown at 37 °C in 10 mL LB medium supplemented with 100 μg/mL ampicillin. The overnight culture was inoculated into one liter LB medium supplemented with 100 μg/mL ampcillin and grown under the same conditions. When cell OD_600_ reached 0.6–0.7, IPTG was added to a final concentration of 0.5 mM to induce the protein expression and cells were further cultured for 4 h in the same condition. The bacterial pellet was suspended in lysis buffer (40 mL, 20 mM Tris-HCl buffer, pH 8.0, containing 100 mM NaCl), followed by a sonication procedure. The mixture was centrifuged at 10,000 g for 30 min. The supernatant was directly loaded onto amylose beads pre-equilibrated with 20 mM Tris-HCl buffer, pH 8.0 containing 100 mM NaCl. The captured MBP-BARc beads were thoroughly washed with the same buffer to remove non-specifically bound proteins and the fusion protein was eluted with the same buffer containing 10 mM maltose. The purity of the MBP-BARc was determined by SDS-PAGE using 12% polyacrylamide gel. The protein concentration was estimated by Bradford method with BSA as the standard.

### 2.4. Removal of the affinity tag and separation of the BARc

The affinity tag in fusion protein MBP-BARc can be cleaved by human rhinovirus 3C protease at the weight ratio of 50:1 (substrate/enzyme) at RT for 20 h. Then, the solid (NH_4_)_2_SO_4_ was slowly added to the reaction mixture until reaching 1 M. The mixture then was centrifuged at 10,000g for 30 min. The tag MBP was kept in the supernatant and the target protein BARc was remained in the precipitate. The BARc precipitate was dissolved with the buffer (50 mM Tris-HCl, 150 mM NaCl, pH7.2) and dialyzed against the same buffer for next step.

### 2.5. Western-blot analysis of BARc domain

Protein samples were run on SDS-PAGE and transferred to nitrocellulose membrane. Non-specific binding sites of the protein were blocked with 1% (w/v) skimmed milk in PBS. The membrane harboring the sample was incubated with guinea pig anti-BARc polyclonal primary antibody and in turn, with the secondary rabbit anti-guinea pig antibody conjugated with horseradish peroxidase (HRP). Finally, the immuno-conjugate was visualized by 3,3’-diaminobenzidine tetrahydrochloride solution [11].

### 2.6. Interaction of PDZ and BARc domains identified by the Pull-down test

An aliquot of Ni^2+^-NTA agarose beads harboring about 20 μg PDZ domain were mixed with different amounts (0, 25, 50, 100 μg) of the soluble BARc domain in total 500 μl buffer and incubated for 2 h at 4 ºC. Each sample was washed five times with 1 mL buffer (50 mM Tris-HCl, 150 mM NaCl, 5mM imidazole, pH7.5) until no protein was detected in the washing solution. Finally, the captured resin was suspended in 20 μL loading buffer and analyzed on SDS-PAGE.

### 2.7. Protein Lipid Overlay (PLO) assay

Serial amounts of bovine brain lipid extracts (200, 20, 2 and 0.2 ng) were spotted onto a nitrocellulose membrane and blocked with 2% (w/v) BSA in PBS. Then, the membrane was incubated with 100 nM MBP, MBP-BARc and BARc at room temperature for 2 h, respectively. After being thoroughly washed with PBS buffer, the protein-lipid complex on the membrane was detected by the immunological assay as described as Western blot analysis.

## 3. Results and Discussion

### 3.1. Expression and purification of fusion protein MBP-BARc

**Figure 2.**
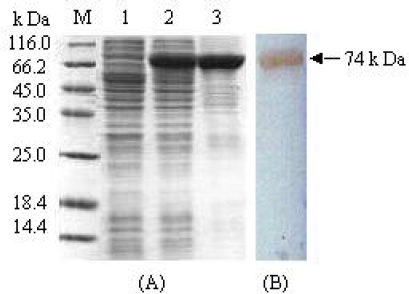
SDS-PAGE analysis and Western blotting of MBP-BARc. (A) SDS-PAGE M: Protein marker; Lane1: un-induced cell lysate; Lane 2: induced cell lysate; Lane 3: product eluted from the amylose column; (B) Western blotting.

Attempting to express a soluble BARc, we constructed different expression vectors involving pET-*barc* with the vector pET32a, pG-*barc* with pGEX-6p-1, pH-*barc* with pHGB and pMAL-s-*barc* with pMAL-s etc. Unfortunately, although each reconstruct could more and less express the corresponding protein MBP-BARc, all of them existed in the precipitate except pMAL-s-*barc* in *E.coli*. In such case, our experiment was locked on the fusion protein, MBP-BARc. It is shown from SDS-PAGE analysis that the cell lysate dominantly reveals a 74 kDa band (Figure 2, A. Lane 2), corresponding 42 kDa MBP plus 32 kDa BARc. The yield of the fusion protein is about 35% of total proteins in cells, as detected by Gel Document Scanning (GDS). The fusion protein, MBP-BARc was further confirmed by Western blot analysis with either anti-MBP monoclonal antibody or anti-BARc polyclonal antibody (Figure 2. B). Based on MBP affinity, the supernatant from cell lysate can be directly loaded on amylose beads. MBP-BARc may be tightly harbored to amylose beads and eluted with the buffer including 10 mM maltose, whilst non-specific proteins were thoroughly washed out by the buffer only. The fusion protein purified is quite pure as shown in Figure 2 (A. Lane3). In the routine experiment, about 40–50 mg target protein could be obtained in 1 L culture by one-step affinity chromatography. It is worthily stressed that not only MBP is as an affinity tag in the fusion expression, but also is effective in facilitating or increasing the soluble expression of most recombinant proteins in *E.coli* [11].

### 3.2. Removal of the affinity tag from MBP-BARc

**Figure 3.**
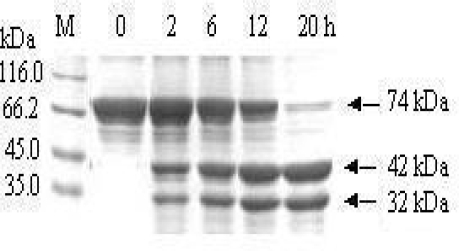
SDS-PAGE analysis for the gradient time test of MBP-BARc cleaved by human rhinovirus 3C protease. M: Protein marker; 0–20 h indicate the reaction time; Labeled bands correspond to MBP-BAR (74 kDa), MBP (42 kDa) and BARc (32 kDa) respectively.

To remove the affinity tag from fusion protein MBP-BARc, an enzymatic cleavage procedure was conducted with human rhinovirus 3C protease under the condition as described in the Materials and Methods section. The result indicated from a gradient time test in Figure 3 that during human rhinovirus 3C protease cleavage, the BARc (about 32 kDa) was gradually released from the fusion product (about 74 kDa) until 20 h or so. The efficiency of removing MBP tag was about 95%, as analyzed by SDS-PAGE (Figure 3). The GDS analysis pointed out that the amount of BARc released from the fusion protein was in direct proportion with human rhinovirus 3C protease cleavage time. The result implies that the human rhinovirus 3C protease, a bench preparation, can efficiently cleave the corresponding tag fused to the target protein.

### 3.3. Separation of the BARc from the human rhinovirus 3C protease reaction mixture

**Figure 4.**
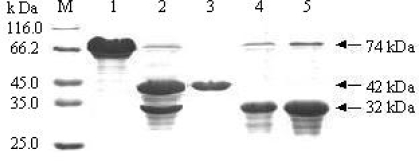
SDS-PAGE analysis of purified MBP-BARc, MBP and BARc. M: Protein marker; Lane 1: MBP-BARc; Lane 2: MBP-BARc cleaved by human rhinovirus 3C protease; Lane 3: MBP in the supernatant of 1.0 M (NH_4_)_2_SO_4_; Lane 4 and 5: re-soluble BARc in the precipitate at 1.0 M (NH_4_)_2_SO_4_.

To separate the BARc from the enzymatic mixture of MBP-BARc, some methods such as ion exchange chromatography, gel filtration and even amylose re-affinity were attempted. Unfortunately, none was successful due to the aggregate of the sample, either BARc or MBP. In such case, we tried a classic method of ammonium sulfate precipitation and unexpectedly found that the BARc could be precipitated at 0.2—1.2 M (NH_4_)_2_SO_4,_ while MBP was still soluble. The phenomenon may mean that the different aggregate state of MBP and BARc results in the different hydration shell round each protein. Finally, we used the concentration of 1.0 M (NH_4_)_2_SO_4_ to successfully separate the BARc from the enzymatic mixture. The BARc purity in the precipitate is about 95%, as analyzed by SDS-PAGE (Figure 4). Although the resoluble BARc may be contaminated with traces of MBP-BARc, it could be used as a sample for its functional tests.

### 3.4. Protein-protein interaction identified by Pull-down test

**Figure 5.**
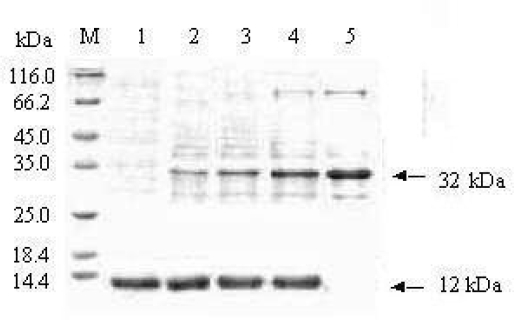
Interaction between PDZ domain and the BARc by the Pull-down test. M: Protein marker; Lane 1: PDZ domain ( 12 kDa); Lane 2–4: the captured PDZ resin was mixed with 25, 50, 100 μg BARc respectively; Lane 5: BARc only (32 kDa).

Pull-down test is based on an immobilized partner which can be combined with its versus ligand. Therefore, the protein-protein interaction may be examined by some methods such as SDS-PAGE, Western blotting, auto-radiography or mass spectrometry and so on [12]. In this report, PDZ-Ni^2+^-agarose prepared by our group was as one partner and the soluble BARc was as versus partner to examine their interaction. It is shown from SDS-PAGE analysis in Figure 5 that the interaction between two proteins reveals two bands, corresponding to BARc (32 kDa) and PDZ domain (12kDa). Moreover, the dose-dependence of BARc concentration (Figure 5 Lane 2—4) has obviously occurred.

### 3.5. Combination of the BARc with lipid by PLO assay

**Figure 6.**
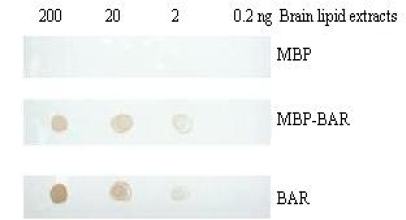
PLO assay for the BARc. The amount of bovine brain lipid extracts was indicated in the top. 100 nM MBP, MBP-BARc and BARc were used respectively.

The Protein Lipid Overlay (PLO) assay is frequently used to detect a special protein combined with lipids [13]. It was reported that PICK1 BAR domain with different lengths could bind to lipids [14]. Here we tried to use lipid extracts from bovine brain to evaluate the binding ability of the BARc. The result shows in Figure 6 that the developing color degree of the BARc is much deeper than that of MBP-BARc, while MBP alone is colorless in the same condition, meaning that the interaction between the BARc and lipid has occurred.

## 4. Conclusions

In conclusion, we report here for the first time that the PICK1 BARc domain can be successfully expressed as MBP-BARc fusion protein in *E.coli*. Cleavage with human rhinovirus 3C protease and subsequent ammonium sulfate precipitation were essential to produce a soluble BARc. This way, the target protein BARc domain could be prepared economically at a higher yield. The recombinant BARc prepared herein could be as a sample to investigate its biochemical function. We are believed that this study may provide a new thought for the protein purification in the field of protein engineering.
